# Effect of Rho-Associated Kinase Inhibitor and Mesenchymal Stem Cell-Derived Conditioned Medium on Corneal Endothelial Cell Senescence and Proliferation

**DOI:** 10.3390/cells10061463

**Published:** 2021-06-11

**Authors:** Boyoung Jung, Hun Lee, Sumi Kim, Hungwon Tchah, Changmo Hwang

**Affiliations:** 1 Department of Convergence Medicine, University of Ulsan College of Medicine, Seoul 05505, Korea; jbobo0616@gmail.com; 2 Biomedical Engineering Research Center, Asan Institute for Life Sciences, Asan Medical Center, Seoul 05505, Korea; sumi0326@naver.com; 3 Department of Ophthalmology, University of Ulsan College of Medicine, Seoul 05505, Korea; yhun777@gmail.com; 4 Department of Ophthalmology, Asan Medical Center, Seoul 05505, Korea

**Keywords:** rabbit corneal endothelial cells, ROCK inhibitor, Y-27632, mesenchymal stem cell-derived conditioned medium, senescence

## Abstract

This study aims to obtain sufficient corneal endothelial cells for regenerative application. We examined the combinatory effects of Rho-associated kinase (ROCK) inhibitor Y-27632 and mesenchymal stem cell-derived conditioned medium (MSC-CM) on the proliferation and senescence of rabbit corneal endothelial cells (rCECs). rCECs were cultured in a control medium, a control medium mixed with either Y-27632 or MSC-CM, and a combinatory medium containing Y-27632 and MSC-CM. Cells were analyzed for morphology, cell size, nuclei/cytoplasmic ratio, proliferation capacity and gene expression. rCECs cultured in a combinatory culture medium showed a higher passage number, cell proliferation, and low senescence. rCECs on collagen type I film showed high expression of tight junction. The cell proliferation marker Ki-67 was positively stained either in Y-27632 or MSC-CM-containing media. Genes related to cell proliferation resulted in negligible changes in MKI67, CIP2A, and PCNA in the combinatory medium, suggesting proliferative capacity was maintained. In contrast, all of these genes were significantly downregulated in the other groups. Senescence marker β-galactosidase-positive cells significantly decreased in either MSC-CM and/or Y-27632 mixed media. Senescence-related genes downregulated LMNB1 and MAP2K6, and upregulated MMP2. Cell cycle checkpoint genes such as CDC25C, CDCA2, and CIP2A did not vary in the combinatory medium but were significantly downregulated in either ROCK inhibitor or MSC-CM alone. These results imply the synergistic effect of combinatory culture medium on corneal endothelial cell proliferation and high cell number. This study supports high potential for translation to the development of human corneal endothelial tissue regeneration.

## 1. Introduction

Human corneal endothelial cells (HCECs) form a monolayer of orderly hexagonal cells that act as a barrier to separate the corneal stroma from the aqueous humor of the anterior chamber. CECs are a nutritional gateway and regulate the water content of the corneal stroma [1,2]. Thus, CECs play an important role in maintaining homeostatic corneal transparency by exerting both pump function mediated via Na^+^/K^+^ ATPase and barrier function facilitated via the tight-junction protein zona occludens-1 (ZO-1) [1,2,3,4].

CECs have a severely limited proliferative ability. They show no functional regeneration in vivo due to contact-inhibition at the G1 phase in the cell cycle [5,6,7,8]. Hence, these cells are not able to replicate after an injury or disease, resulting in a low density of cells and loss of pump and barrier function [7]. CECs can be damaged during corneal endothelial dystrophy, ophthalmic surgery, and trauma. The number of CECs also decreases with age. Cells obtained from older donors grow slower, exhibit a higher heterogeneity, and are more prone to senescence compared to those derived from younger donors [6]. If cell density decreases below a critical level (500 cells/mm^2^ cell density), the corneal physiological function fails and corneal edema ensues, eventually leading to bullous keratopathy and loss of visual acuity. In the case of terminal conditions that do not respond to treatments, endothelial keratoplasty is the only option for reversing this corneal endothelial damage. However, a lack of suitable donor tissue for corneal transplantation and problems associated with surgery, including allograft rejection, primary graft failure, and continuous loss of cell density, have yet to be resolved.

Although researchers have tried to expand the number of primary CECs isolated from human donor tissues in vitro, massive production of CECs from a small number of healthy, qualified donor tissues remains challenging due to their limited regenerative capacity [7,9]. Rho-associated kinase (ROCK) mediates various cellular events, such as proliferation, differentiation, apoptosis, and oncogenic transformation [10]. A selective ROCK inhibitor, Y-27632, exhibits unique effects on cultured CECs, including proliferation promotion, cell adhesion enhancement, and apoptosis suppression [11,12]. Application of Y-27632 as eye drops also promotes CEC proliferation and enhances corneal endothelial wound healing in rabbit and monkey in vivo models [13,14]. Y-27632 eye drops recover corneal clarity and thickness in some patients with focal-edema-type Fuchs’ corneal dystrophy [13,15]. Mesenchymal stem cells (MSCs) exert paracrine effects via the secretion of growth factors, cytokines, and other substances. Bone marrow MSC-derived conditioned medium (MSC-CM) promotes the proliferation of HCEC by regulating the G1 proteins of the cell cycle and maintains functional proteins of the CEC such as ZO-1 and Na^+^/K^+^-ATPase [16,17]. Similarly, MSCs derived from umbilical cord blood can “home” to injured corneal endothelium tissues and can be differentiated into HCEC-like cells [18]. Considering the positive effects of Y-27632 and MSC-CM on CECs, we aimed to investigate the combinatory effects of ROCK inhibitor Y-27632 and MSC-CM on the senescence and proliferation of rabbit CECs.

## 2. Materials and Methods

### 2.1. Materials

Phosphate buffer saline (PBS), Dulbecco’s Modified Eagle’s medium (DMEM), fetal bovine serum (FBS), trypsin-EDTA (TE), antibiotic-antimycotic (AA), Alexa Fluor 594 goat anti-mouse IgG, mouse monoclonal antibody (mAb) against ZO-1, Rhodamine phalloidin, Alexa Fluor 488 goat anti-rabbit IgG and AlamarBlue Cell Viability Reagent were purchased from Life Technologies Korea (Seoul, Korea). Y-27632 was purchased from Sigma-Aldrich (St. Louis, MO, USA), and collagen film was obtained from Bioland (Seoul, Korea). A senescence β-galactosidase staining kit was purchased from Cell Signaling Technology (Beverly, MA, USA). Anti-Ki-67 (Cat. No: ab6526) and anti-caspase-3 (Cat. No: ab52181) were obtained from Abcam (Cambridge, UK), and anti-Na^+^/K^+^ ATPase (SC-21712) was obtained from Santa Cruz Biotechnology (Santa Cruz, CA, USA).

### 2.2. Primary Culture of Rabbit Corneal Endothelial Cells

Rabbit CECs(rCECs) were isolated from adult New Zealand white rabbits, with weights ranging from 1.8 to 2.2 kg (Orient Bio Inc., Seongnam-si, Gyeonggi-do, Korea). Corneal endothelium was scraped without removing the Descemet’s membrane. After incubation for five minutes at 37 °C in 0.25% TE digestion, rCEC suspension was collected, centrifuged (2000 rpm: 800× *g*) for five minutes, and cultured on a Laminin-521 (ThermoFisher Scientific, Cat. No.: A29249)-coated cell culture plate with CEC basal medium of DMEM containing 20% FBS and 1% AA. The rCECs were cultured in a humidified atmosphere at 37 °C under 5% CO_2_, and the rCEC culture medium was replaced every two days. When the cells were confluent, a serial passage was performed at a ratio of 1:3. The cell number for each passage was 2.1 × 10^4^ cells/cm^2^. In the early stages of rCEC culture (passage 2), the cell number was confirmed by directly counting cells with trypan blue staining and a hemocytometer.

### 2.3. Preparation of Human Umbilical Cord-Mesenchymal Stem Cell-Derived Conditioned Medium (CM)

Human umbilical cord‒derived MSCs (UC-MSCs) were obtained from the Stem Cell Center (Asan Medical Center, Seoul, Korea). UC-MSCs at passage numbers 5–7 were used for CM collection. UC-MSCs were cultured in DMEM with 5% FBS and 1% AA. After UC-MSCs reached 80–90% confluence, rabbit CEC culture medium was added to UC-MSC cultured dishes after gentle rinsing with PBS. The UC-MSCs were further cultured for 24 h, and CM was collected, centrifuged at 2000 rpm (800× *g*) for 10 min, filtered through a 0.2 μm syringe filter, stored at 4 °C, and used within seven days.

### 2.4. Determination of Rho-Associated Kinase Inhibitor Concentration and Mesenchymal Stem Cell-Conditioned Media Volume Ratio

**Figure 1:**
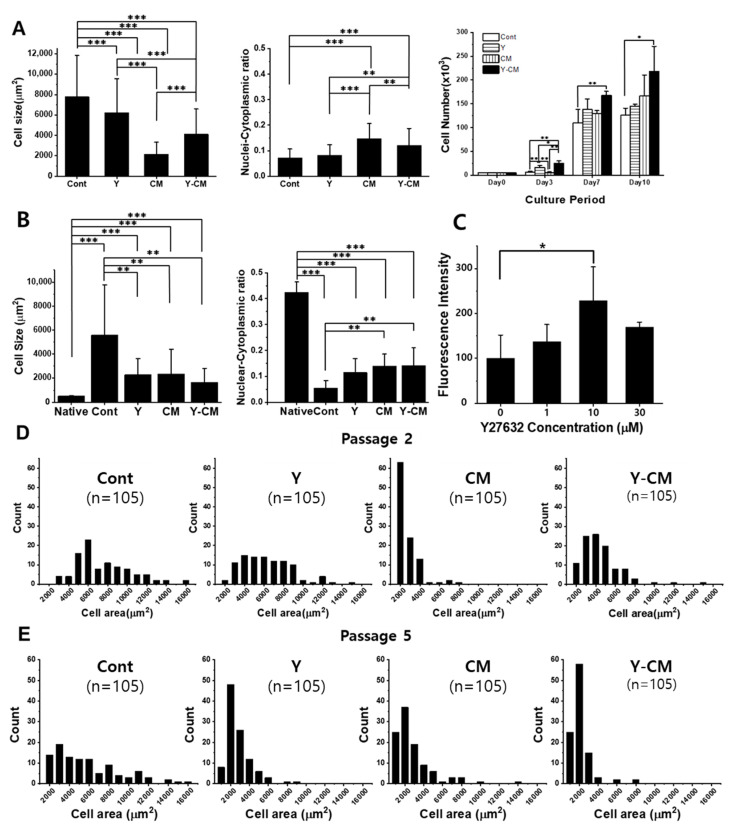
Morphological analysis of rCECs at passage numbers 2 and 5. (**A**) Cell size and nuclei-cytoplasmic ratio (n = 20), and cell number change during 10-day culture with AlamarBlue assay of passage number 2. (**B**) Cell size, nuclear-cytoplasmic ratio (n = 20) of native tissue and passage 5. (**C**) Optimization of Y-27632 concentration with rCEC passage 2. (**D**,**E**) Cell size distribution of passage 2 and passage 5 cultured for seven days (n = 105). (mean ± SD, * *p* < 0.05, ** *p* < 0.01, *** *p* < 0.001).

To optimize the concentration of ROCK inhibitor, CECs were cultured with a different concentration of Y-27632. We obtained fluorescence of AlamarBlue cell proliferation/cytotoxity assays after rCECs were cultured for 24 h in 0, 1, 10, and 30 μM Y-27632 in a basal culture medium. The Y-27632 concentration of 10 μM showed the highest cell proliferation and was chosen for further study (Figure 1D) [19].

The mixing ratio of MSC-CM in CM-containing groups was chosen from previous studies, and a 1:1 volume ratio of basal medium: MSC-CM was used throughout this study [20].

### 2.5. CEC Passage Number under Different Cell Culture Media

Primary cultured rCECs were used throughout this study unless otherwise noted. rCECs were cultured under four different conditions by combining basal medium, CM and Y-27632. These experimental groups included a control group (basal medium: DMEM+20% FBS+1% AA), Y group (basal medium+10 μM Y-27632), CM group (basal media:CM (1:1)), and a Y-CM group (basal media:CM (1:1)+10 μM Y-27632). Subsequent cell passage was performed after confluency. For CEC harvesting, cells were treated with a 0.25% trypsin/EDTA solution (TE) for five minutes. The number of cells was counted with trypan blue and a hemocytometer. The cell suspension was loaded on a six-well plate with a cell concentration of 2.1 × 10^5^ cells/cm^2^ for subsequent culture.

### 2.6. Effect of CEC Culture Media on Cell Proliferation

rCECs passage 2 were cultured under four cell culture medium groups as mentioned above. rCEC proliferation was indirectly measured via an AlamarBlue Cell Viability test on days 1, 3, 7, and 10 according to the manufacturer’s protocol. In brief, rCECs were incubated in the culture media mixed with 10% (*v*/*v*) AlamarBlue Cell Viability Reagent and incubated for three hours. The fluorescence intensity of 570 nm and 600 nm was measured using a fluorescence microplate reader (Victor^TM^ X2, PerkinElmer, MA, USA). The fluorescence values of the known number of cells at day 0 were used as the cell number calibration.

### 2.7. Senescence-Related β-Galactosidase Activity Staining

Rabbit CECs were seeded at a density of 2 × 10^4^ cells/cm^2^ on 12-well plates, cultured for 7 days under each condition, and stained using a senescence β-galactosidase staining kit per the manufacturer’s instructions (Cell Signaling Technology). In brief, cultured CECs were washed with PBS once and fixed with a fixative solution for 10 min at RT. Then, they were washed twice with PBS and stained with a staining solution mix to examine the senescence-related β-galactosidase (SA-β-gal) activity at 37 °C in a dry oven overnight. Stained samples were observed under a microscope at RGB mode (EVOS FL Auto, Life Technologies Korea, Seoul, Korea). SA-β-gal-positive cells were manually counted with ImageJ software (*n* = 3 images).

### 2.8. Immunofluorescence Staining

Samples were fixed in cold methanol for 5 min. Subsequently, they were treated with Triton X-100 for 30 min, washed with PBS, and blocked for 1 h in 3% human serum albumin mixed with PBS before antibody conjugation. Anti-Ki67 (1:100; proliferation marker), caspase-3 (1:100; apoptosis marker), ZO-1 (1:100; tight junction marker), and Na^+^/K^+^ ATPase (1:100, pump function marker) were used as the primary antibodies, and Alexa Fluor anti-rabbit 488 (Molecular Probes) or anti-mouse 594 were used as secondary antibodies. Nuclei were counterstained with DAPI. Stained samples were observed under a fluorescence microscope (EVOS FL Auto).

### 2.9. Cell Size and Nuclei-Cytoplasmic Ratio Measurement

rCECs in passage 2 and passage 5 were measured for cell size and nuclei-cytoplasmic (N/C) ratio. Cell and nuclei size were measured from phase contrast and immunofluorescence microscope images using ImageJ software. Individual cell and nuclei size were measured by binary conversion particle counting function. A total of 105 cells were randomly measured from each group for cell size. The nuclei-cytoplasmic ratio was obtained from 20 cells in each group.

### 2.10. Effect of Culture Media Changes on rCECs at a High Passage Number

rCECs cultured up to passage 7 in Y-CM medium were subcultured and the culture media was changed at passage 8 to observe the effect of culture media at high passage number. rCECs underwent AlamarBlue assay for cell proliferation/cytotoxicity at culture day 4. Cell morphologies were observed with an optical microscope on culture day 7.

### 2.11. Effect of Scaffold on ZO-1, Na^+^/K^+^-ATPase Cell Functional Markers

rCECs passage 2 were cultured on a collagen film with different media conditions. Cells were immunostained to observe corneal endothelial functional markers ZO-1 and Na^+^/K^+^-ATPase.

### 2.12. RNA Sequencing

The rabbit CEC sample used for the initial state of passage 1 was designated as a positive control. The samples for the control and Y groups were prepared at passage 3, as the cell proliferation rate in these two groups significantly decreased after this passage. For the CM and Y-CM groups, the rCECs were proliferated beyond passage 5, where the RNA was prepared at passage 5 for these two groups. The seed total RNA was extracted using a modified version of the CTAB method. For quality control, a NanoDrop spectrophotometer (Thermo Fisher Scientific Inc., Waltham, MA, USA) and Agilent 2100 Bioanalyzer (Agilent Technologies, Santa Clara, CA, USA) were used to determine the quantity, quality, and reliability of the total RNA. Samples with an RNA integrity number (RIN) of approximately 8.0 or higher were selected for library preparation and sequencing. The mRNA library preparation was performed using the SureSelect Strand-Specific RNA Library Prep required for Illumina Multiplexed Sequencing (protocol version E0, March 2017). Consequently, RNA sequencing was performed using Illumina HiSeq™ 4000 (Theragene Etex, Seoul, Korea).

### 2.13. Statistical Analysis

Statistical analyses were performed using OriginPro 8.0 software (Northampton, MA, USA). Results are expressed as means ± standard deviation. A means comparison between the two groups was performed using one-way ANOVA and Tukey test. A *p* value less than 0.05 was considered statistically significant.

## 3. Results

### 3.1. rCEC Passage Number, Cell Proliferation and Morphology

rCECs of the Cont and Y groups could be cultured up to P5. The CM and Y-CM cell number ratio increased by more than two times that of Cont and Y at P2. CM and Y-CM could be cultured up to P6 and P10, respectively.

The cell number of the Cont and Y groups increased, but less than 10 times the initial seeding number at P2, and they eventually decreased at P5. In contrast, rCECs in the CM and Y-CM group showed increased cell numbers up to P6 and P10 (data not shown).

**Figure 2:**
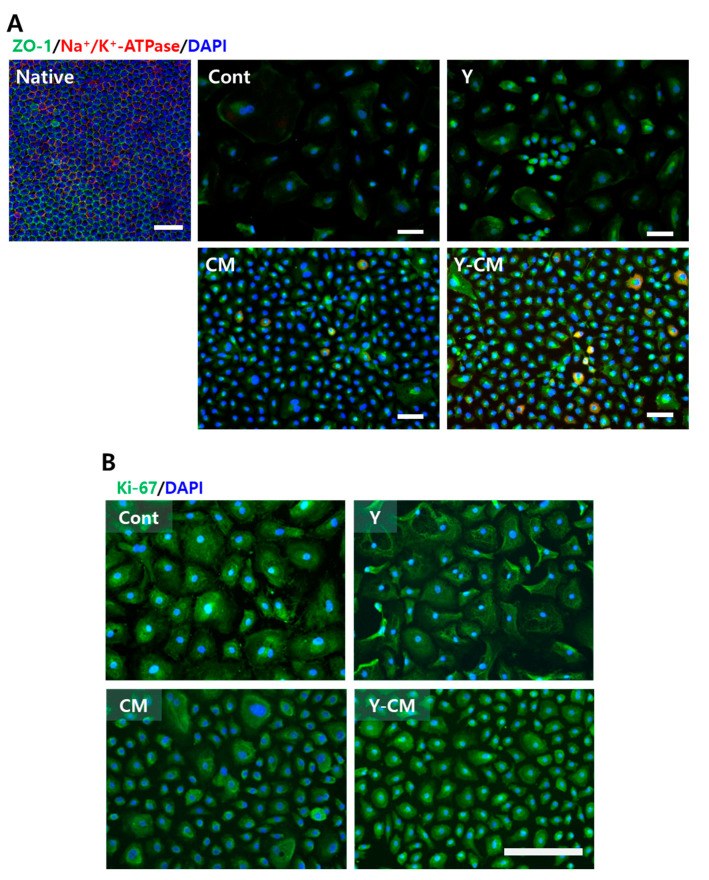
Immunofluorescence images of native tissue and in vitro cultured rCECs at passage number 2 culture day seven. (**A**) Function-related markers, ZO-1 (green), Na^+^/K^+^-ATPase (red). Native: native rabbit corneal endothelial tissue. (**B**) Cell proliferation marker Ki-67 (green) for cell proliferation. Nuclei were stained with DAPI (blue). (Scale bar = 100 μm).

Morphological observations with a microscope revealed rCECs of the Cont group with irregular shapes in P2 (Figure 2). rCECs of P2 in CM demonstrated the smallest cell size (Figure 1A). Cells cultured with ROCK inhibitor (Y, Y-CM) had a relatively polygonal structure in P2.

### 3.2. Cell Size and the Nuclei-Cytoplasmic Ratio of rCECs

In vitro cultured rCECs at passage number 2 (P2) and passage 5 (P5) showed a significantly higher cell size than native rCECs in all conditions (Figure 1A,B).

The cell areas of native rCECs were 521.3 ± 48.8 μm^2^, whereas the P2 cell sizes were Cont 7781.8 ± 4052.3 μm^2^, 6213.7 ± 3341.2 μm^2^, 2170.8 ± 1148.1 μm^2^, and 4112.0 ± 2476.2 μm^2^, respectively, and P5 rCECs were 5579.9 ± 4167.5 μm^2^, 2290.1 ± 1341.4 μm^2^, 2337.9 ± 2057.8 μm^2^, and 1653.8 ± 1163.2 μm^2^ (mean ± SD) for the Cont, Y, CM, and Y-CM groups, respectively (Figure 1A,B). All the in vitro cultured P2 and P5 cells have significantly larger mean values than native rCECs, showing at 1490%, 1192%, 416%, 789% for P2 and 1070%, 439%, 448% and 317% for P5 for Cont, Y, CM, and Y-CM, respectively (*p* < 0.001). Among the in vitro cultured P2 and P5 rCECs, cells in the control medium have significantly higher cell size at a significance of *p* < 0.01 compared to all the other groups. There was a significant difference in in vitro cultured cell area among P2 rCECs (*p* < 0.01), whereas there was no statistical difference among Y, CM, and Y-CM groups for P5 cells. A cell area distribution histogram of in vitro cultured rCECs is shown in Figure 1D,E. P2 rCECs peaked at medium value 6000 μm^2^, 4000 μm^2^, 2000 μm^2^, and 3000 μm^2^, respectively. Among P5 rCECs, the Cont group had a peak in median value at 3000 μm^2^, whereas other groups show the highest peak at a median value of 2000 μm^2^.

As shown in Figure 1A,B, native rabbit corneal endothelium tissue cells had a nuclei-cytoplasmic ratio (N/C ratio) of 0.42 ± 0.04. The N/C ratios of P2 rCECs were 0.072 ± 0.034, 0.082 ± 0.042, 0.15 ± 0.058 and 0.12 ± 0.067, and the N/C ratios of P5 cells were 0.055 ± 0.028, 0.11 ± 0.054, 0.14 ± 0.045, and 0.14 ± 0.069 for Cont, Y, CM, and the Y-CM groups, respectively (*p* < 0.001).

### 3.3. Immunofluorescence Staining Proliferation Marker and Tight Junction Marker

The staining of the tight junction marker ZO-1 was dispersed on the plasma membranes, not localized in the cell–cell contact region in all groups both P2 and P5. Na^+^/K^+^ -ATPase marker, an indicator of pump function, was expressed in only a few cells in the CM and Y-CM groups when cultured on a culture plate (Figure 2A). Immunofluorescence images showed substantially higher expression of the proliferation marker Ki-67 in the CM and Y-CM groups than the Cont and Y groups (Figure 2B). Cell apoptosis marker caspase-3 had no significant expression in all groups at P2 (Supplementary Figure S1).

### 3.4. Cell Morphology on Collagen Film

**Figure 3:**
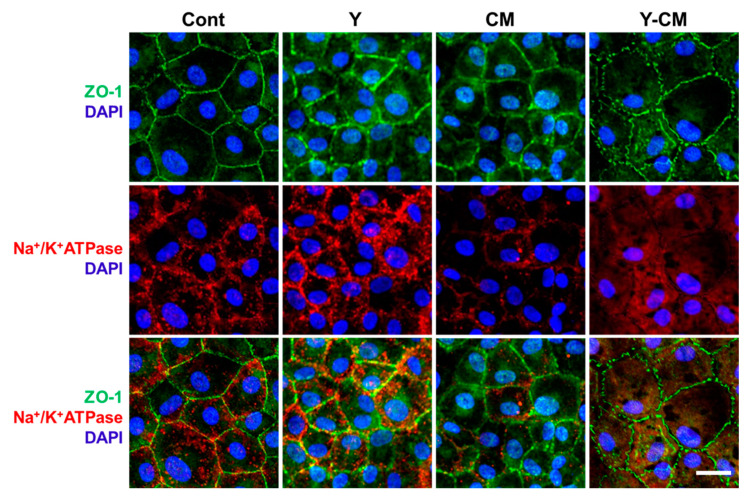
Confocal microscopy of P2 rCECs cultured on collagen film. ZO-1 (green), Na^+^/K^+^-ATPase (red) and DAPI (blue) (Scale bar = 50 μm).

Figure 3 shows confocal microscopy images of P2 rCECs on collagen film. Cells express polygonal shape, the tight junction marker ZO-1 and pump marker Na^+^/K^+^-ATPase. The tight junction marker ZO-1 was expressed along the border of cell–cell contact. Further, Na^+^/K^+^-ATPase exhibited relatively strong expression on the collagen film as compared to the well plate (Figure 3). The expression of ZO-1 was different among all of the groups. The Y-CM group has a ZO-1 marker immature tight junction, which appears as dotted lines along cell–cell contact boundaries, whereas it is expressed as connected lines in Cont group (Figure 3). Expression of Na^+^/K^+^-ATPase was aggregated in Cont, Y and CM groups, but Y-CM group had a distributed configuration.

### 3.5. Senescence

**Figure 4:**
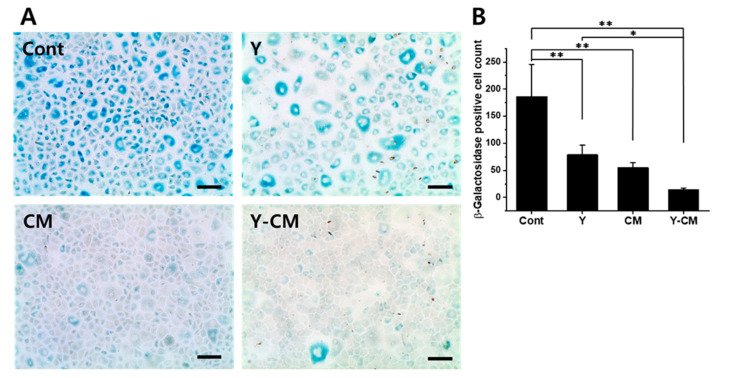
Senescence assay of seven-day cultured rCECs at passage number 2. (**A**) β-galactosidase staining (scale bar = 400 μm). (**B**) β-galactosidase staining positive cell number count. (mean ± SD, n = 5, * *p* < 0.05, ** *p* < 0.01).

The senescence marker SA-β-gal activity was stronger in the Cont and Y groups than in the CM and Y-CM groups (Figure 4A). The number of SA-β-gal-positive cells was significantly lower in the CM and Y-CM groups compared to that observed in the control group (Figure 4B).

### 3.6. Morphology and Effects of Media Exchange at High Passage Number

**Figure 5:**
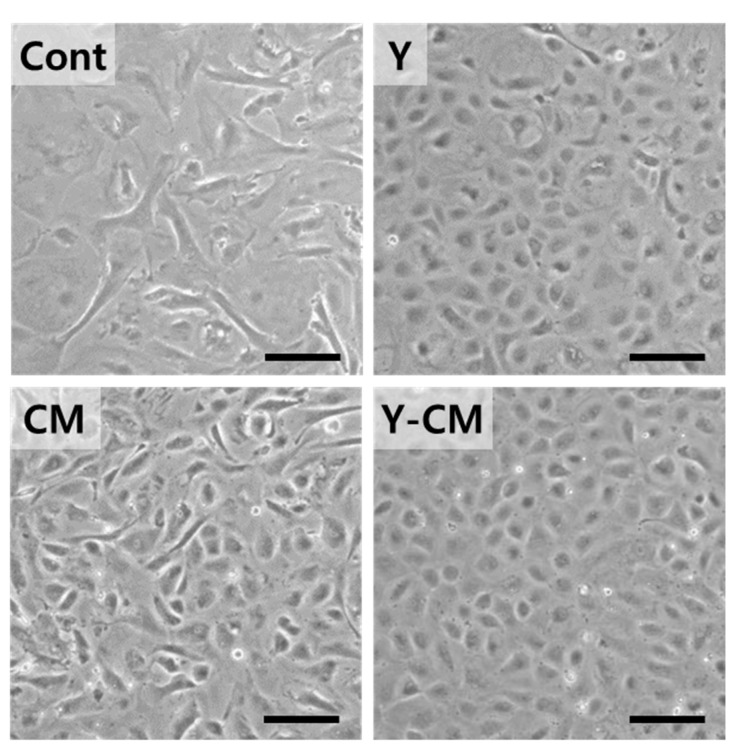
Phase contrast image of in vitro cultured rCECs with passage number 5 on culture day seven (Scale bar = 200 μm).

Morphological observation at high passage number P5 showed that Cont group showed irregular shapes, whereas cultured with ROCK inhibitor (Y, Y-CM) had a relatively polygonal structure (Figure 5). Cells in CM have more fibrotic and mixed with a small portion of polygonal cells at P5 (Figure 5). Y-CM retain polygonal morphology and smallest cell size at P5 (Figure 1B and Figure 5. However, the configuration of rCECs in Y-CM changed to fibroblastic at P10 and were not cultured any further (data not shown).

**Figure 6:**
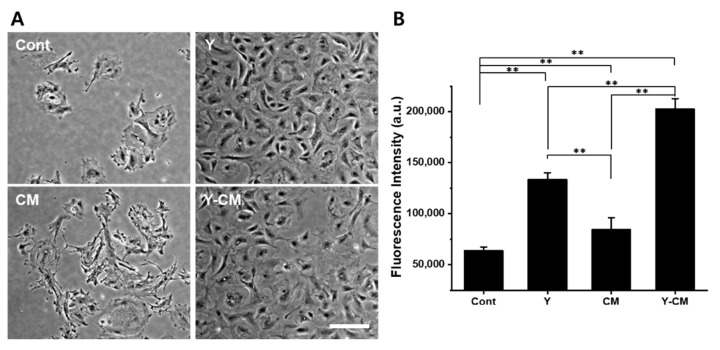
Cell culture medium change’s effect on cell morphology and cell proliferation at high passage number. rCECs were cultured in Y-CM up to passage number 7 and cultured in a different culture media at passage number 8. (**A**) Phase-contrast image of rCECs on culture day seven. (**B**) AlamarBlue assay for mitochondrial activity on culture day day. (mean ± SD, n = 3, ** *p* < 0.01).

When cells cultured up to passage 7 with Y-CM were seeded for passage 8, the culture media was rearranged with four different media for Cont, Y, CM, and Y-CM. On day one after incubation, cells cultured in Cont and CM media exhibited very poor adherence to the substrates. Cells in Y and Y-CM had similar cell morphology to the cells of P7 in Y-CM (Figure 6A). The AlamarBlue assay revealed that the fluorescence intensity of Y and Y-CM were more than two times that of Cont and CM (Figure 6B).

### 3.7. RNA Sequencing

**Figure 7:**
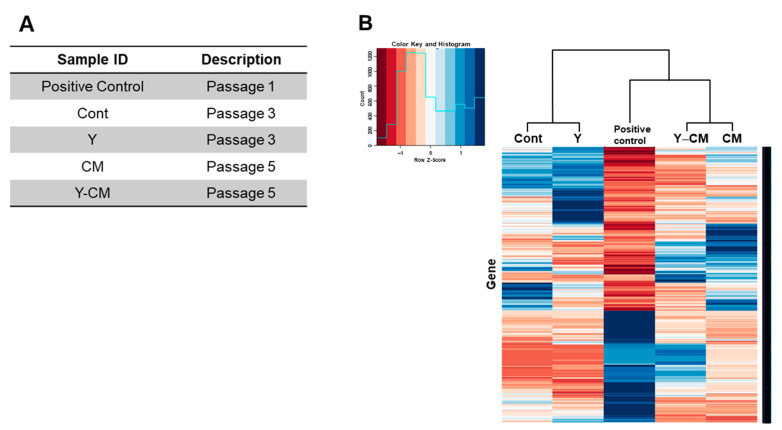
RNA sequencing in RCECs of different culture conditions in vitro (**A**) Sample list of rCECs. (**B**) Clustergram analysis of gene expression.

Figure 7A denotes the passage number of rCECs for RNA sequencing for each cell culture medium group. Clustergram analysis of gene expression in Figure 7B shows a similarity between the positive control and Y-CM groups compared to the other groups.

**Figure 8:**
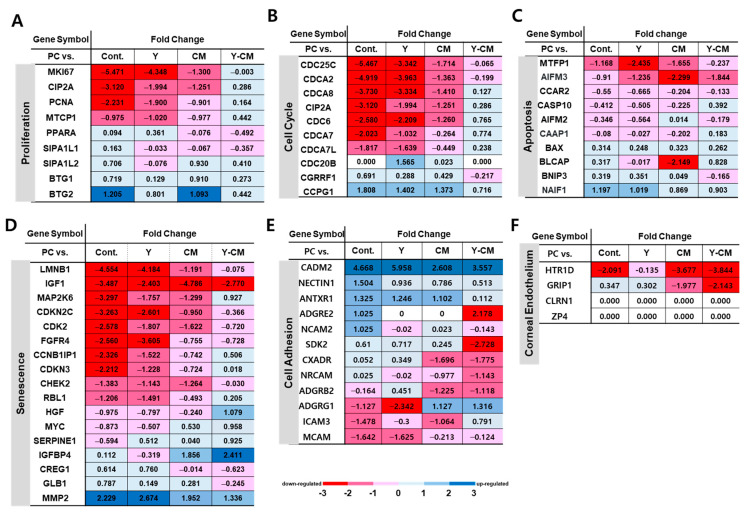
RNA sequencing result of rCEC passage number 2 at culture day seven. Fold change of (**A**) proliferation. (**B**) cell cycle, (**C**) apoptosis, (**D**) senescence, (**E**) cell adhesion, (**F**) corneal endothelial cell marker-related genes.

Gene expression of cell proliferation, cell cycle, senescence, apoptosis, cell adhesion, and CEC markers is summarized in Figure 8. Among cell proliferation genes, MKI67, CIP2A, PCNA and MTCP1 genes were different between positive control and Y-CM groups. MKI67 is significantly decreased in the Cont (−5.471), Y (−4.348), and CM (−1.300) groups, whereas the Y-CM group shows minor changes (−0.003) with respect to P1-positive control rCECs (Figure 8A).

Cell cycle-related genes are listed in Figure 8B. CDC25C, CDCA2, CDCA8, CIP2A, CDC6, CDCA7 and CDCA7L showed decreased expression, and CCPG1 showed positive expression. The Y-CM group showed the least changes in most of the genes listed in Figure 8B.

Senescence-related genes are summarized in Figure 8C. LMNB, IGF1, MAP2K6, CDKN2C, COL1A1, CDK2, FGFR4, CCNB1IP1, and CDKN3 showed a more than two-fold down-regulation of gene expression in the Cont group. Y-CM rCECs showed decreased gene expression of IGF1 and COL1A1 by 2.77 and 1.66 folds, respectively, and IGFBP4 increased by 2.4-fold, as shown in Figure 8C.

Apoptosis-related genes did not change as much as other genes. In the Cont group, only MTFP1 decreased by 1.168-fold and NAIF1 increased by 1.197-fold. Y-CM group showed a 1.844-fold decrease in gene expression in AIFM3, while other genes had a less than one-fold change (Figure 8D).

## 4. Discussion

This study investigated the combinatory effects of a selective ROCK inhibitor, Y-27632, and MSC-CM on rabbit CECs proliferation and senescence. We observed that the combination of these two factors exhibited positive effects on rCEC proliferation, senescence and function conservation in rCECs. Cell culture media mixed with both Y-27632 and MSC-CM had a significantly increased N/C ratio, high proliferation capacity, and decreased senescence. Corneal endothelial-specific marker genes, expression of CLRN1, HTR1D, GRIP1, and ZP4 were confirmed in RNA-seq data, which implies the cells employed in this study were CECs (Figure 8F) [21].

### 4.1. Effect of Media Changes at a High Passage Number

When CECs were cultured in Y-CM up to passage 7 and the culture medium was changed at passage 8, cells in the Cont and CM groups showed poor proliferation and degenerated cell morphology and cells in Y and Y-CM retained their morphology and had a proliferation similar to passage 7 (Figure 6). rCEC culturing in media with Y-27632 and MSC-CM may achieve a high passage number.

### 4.2. Cell Culture on Collagen Film

rCECs cultured on collagen film exhibited ZO-1 and Na^+^/K^+^-ATPase in all groups, which is different from cells cultured in the well plates (Figure 3). Cells on collagen film showed different ZO-1 and Na^+^/K^+^-ATPase distribution. Cont shows clear ZO-1 lines, and Y-CM has immature lines along the cell–cell contact boundaries. This may be explained by the inverse correlation between ZO-1 expression and cell cycling. In Cont group, overexpression of ZO-1 reduces a cell cycle-related proprotein nuclear CDK4 (cyclin-dependent kinase 4) [22], which results in suppression of cell mitosis and cell proliferation. Furthermore, phase separation of ZO proteins derives tight junction formation [23]. Consequently, when ZO-1 is in high concentrations in a cell, cell cycle is arrested, ZO-1 is phase separated, and tight junction forms (Cont). Cells with an underdeveloped tight junction may have low ZO-1 level, and active and proliferative cell cycles (Y-CM). Similar results were observed with corneal endothelial cells cultured on the collagen–vitrigel membrane [24,25].

Na^+^/K^+^-ATPase protein subunits interacts with contactin-associated protein 1(CASPR1), which is located in basolaterial membrane [26,27]. When CECs on collagen film reach confluency, tight junction proteins including ZO-1 and CASPR1 localize along the cell–cell contact lines. As a consequence, CASPR1 interacting Na^+^/K^+^-ATPase pattern forms along the CECs’ borderlines (Figure 3). In contrast, when CECs in Y-CM do not reach confluency and immature tight junction, and Na^+^/K^+^-ATPase is dispersed in the basolaterial membrane, there are no clear lines in immunofluorescence image.

The CECs cultured on laminin-521-coated dishes did not form tight junctions but cells on collagen film did. There exist differences of cell microenvironments—stiffness of substrate (hard vs. soft), extracellular materials (laminin vs. collagen), impermeable culture dish vs. permeable collagen film, or different confluency—which may affect tight junction formation. Further study is warranted to identify the underlying mechanisms of tight junction formation with substrate conditions in CECs.

### 4.3. Senescence

Both Y-27632 and MSC-CM-containing cell culture media have a synergistic effect on senescence-related gene expression levels. The number of SA-β-gal-positive cells and senescence significantly decreased when rabbit CECs were cultured with Y-27632 and MSC-CM combination media. Considering that HCECs derived from older donors are more prone to senescence compared to those obtained from younger donors, a combination of Y-27632 and MSC-CM might maintain their hexagonal shape and endothelial function without inducing senescence [28,29].

When LMNB1, a Lamin B1-related gene, is down-regulated, it is associated to senescence and arrest of cell proliferation. Only Y-CM showed less down-regulated level of LMNB1, which implies least change in cell proliferation ability compared to positive control. As shown in Figure 8C, MMP2 is up-regulated and mRNA of multiple MMPs is reported up-regulated in senescent cells, which may explain the MMP2 gene expression data in Figure 8 [30,31,32]. Similar to proliferation and cell cycle gene expression data, the Y-CM group demonstrates a synergistic effect on reducing cell senescence in culture media compared to Y or CM groups.

### 4.4. Cell Cycle

Among the cell cycle-related genes, CDC25C activates the CDK genes, which is the control checkpoint of cell cycle phases, including entry into mitosis and the S phase. CDC25C reportedly encodes proteins that direct dephosphorylation of cyclin B-bound CDC2 and trigger mitosis [33]. CDC25C gene expression is −5.467-fold down-regulated in Cont group (Figure 8B); under this condition cells may lose mitotic ability. This may explain the poor cell number increase of Cont in P2. The addition of either Y-27632 or MSC-CM substantially reduced the cell cycle-related gene expression. This result may partially explain why the treatment of rabbit CECs with 10 μM Y-27632 under in vitro culture conditions promoted cell adhesion and inhibited apoptosis but did not promote cell proliferation. Y-27632 treatment may have mediated a delay in the G1 to S phase transition [34,35]. In contrast, the Y-CM group showed the smallest change in the CDC25C gene, which drives the cell cycle process (Figure 8B). It also suppresses p53-induced growth arrest [12]. Down-regulation of cell cycle genes means that cells have lost trigger into mitosis and consequently showed decreased proliferation ability. The reduction in the down-regulated cell cycle genes and proliferation genes of both means that the cells have the least changes in cell proliferation ability in comparison to highly down-regulated cells in Cont, Y and CM groups. In our study, only the Y-CM cell culture medium conserved genes supporting cell cycle progress and mitosis.

### 4.5. Apoptosis

RNA sequencing data shows two apoptosis-related genes causing a more than one-fold change in the Cont group. This result indicates that there is no significant expression of the apoptosis in the culture conditions employed in this study. This result coincides with the immunostaining results indicating no significant staining of apoptosis marker caspase-3 in Supplementary Figure S1.

rCECs used for RNA sequencing are P3 for the Cont and Y groups and P5 for CM and Y-CM groups. Despite the high passage number, Y-CM-conserved gene expression enhances cell proliferation and the cell cycle, reducing senescence.

### 4.6. ROCK Inhibitor Y-27632 and MSC-CM Effects

Y-27632 reportedly enhances cellular adhesion and rCEC proliferation [12,36] and proliferation of human CECs in vitro [36]. The ROCK inhibitor upregulates cyclin D and down-regulates p27^kip1^ (p27) via PI 3-kinase signaling, which subsequently promotes G1/S progression in CECs [37].

Several reports have been published on MSC exosomes, such as bone marrow-derived endothelial progenitor cell-conditioned medium [17], bone marrow mesenchymal stem cell-conditioned medium [38], mouse embryonic stem cell-conditioned medium [20], and human umbilical cord mesenchymal stem cell-derived conditioned medium [35]. Conditioned medium contains exosomes which stimulate rat CEC proliferation and maintain functional markers, such as ZO-1, aquaporin 1, and Na^+^/K^+^- ATPase [38,39,40,41]. Bone marrow-derived MSC-CM promoted the proliferation of HCECs by regulating G1 proteins and helped the HCECs maintain functional protein phenotypes, which were localized at the intercellular adherent junctions [16]. MSC-CM contains extracellular vesicles (MSC-EVs) with biological activities, expressing ER stress targeting miRNAs [39], inducing proliferation and survival of human CECs [40,41].

The major aim of this study was to achieve sufficient number of CECs to resolve cell shortage. Achieving high passage number to harvest enough number of CECs, and conserving cell functionalities such as polygonal morphology, tight junction, and cell functional markers would be essential steps for corneal endothelium regeneration. Unfortunately, the conditions employed in this study showed feasibility in extending cell passage with higher cell number, but we could not maintain cell morphology with functionality. Maintaining corneal endothelial cell morphology and functional markers still needs further studies prior to clinical translation.

In summary, the combination of ROCK inhibitor and MSC-CM has synergistic effects on cell proliferation, the cell cycle progress, and low senescence.

The combinatory effects of the ROCK inhibitor and MSC-CM for rCEC culture medium and collagen film substrate may be applied to CEC-based corneal tissue regeneration. Further research is warranted for human-derived CECs and to optimize scaffold materials for clinical translations in the future.

## 5. Conclusions

We demonstrated that the combination of Y-27632 and MSC-CM exerted synergistic effects on senescence and proliferation better than separated applications. Y-CM showed decreased senescence, increased proliferation capacity, and the expression of functional markers, which confirmed gene expression. Further, rCECs on a collagen scaffold showed polygonal morphology and tight junctions. These results suggest that the combination of Y-27632 and MSC-CM may serve as an efficient tool for translating HCECs’ expansion to treat corneal endothelial damage and promote regenerative medicine.

## Data Availability

Data are available upon request from the authors.

## Supplementary Materials

The following are available online at https://www.mdpi.com/article/10.3390/cells10061463/s1, Figure S1: Immunofluorescence images of native tissue and in vitro cultured rCECs at passage number 2 culture day 7.
