# Supplementary material for: Effect of Rho-Associated Kinase Inhibitor and Mesenchymal Stem Cell-Derived Conditioned Medium on Corneal Endothelial Cell Senescence and Proliferation
Source: Cells. 2021 Jun 11;10(6):1463. doi: 10.3390/cells10061463 (PMC8230597; doi:10.3390/cells10061463)
Supplement: Supplementary file 1 [file cells-10-01463-s001.zip › cells-1127884-supplementary.pdf]

Effect of Rho-Associated Kinase Inhibitor and Mesenchymal Stem Cell-Derived Conditioned Medium on Corneal Endothelial Cell Senescence and Proliferation

Supplmentary Figure:

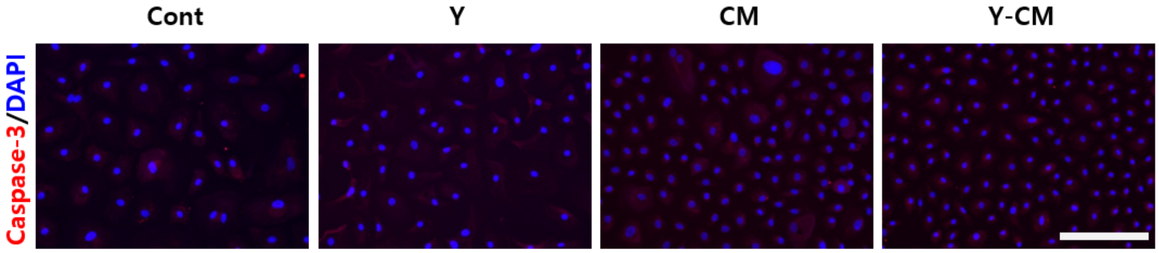

Figure S1. Immunofluorescence images of native tissue and in vitro cultured rCECs at passage number 2 culture day 7. Caspase-3 (red) for apoptosis. Nuclei were stained with DAPI (blue) (Scale bar = 100 μm).
